# Hemispheric H3K27M-mutant diffuse glioma in an adult: a rare entity with ATRX loss and Oligodendroglioma-like features

**DOI:** 10.1093/omcr/omag063

**Published:** 2026-05-10

**Authors:** Ghaida S AlSugair, Mohammad Anas Dababo

**Affiliations:** Department of Basic Sciences, College of Medicine, Princess Nourah bint Abdulrahman University, King Khalid Airport Road, Riyadh 11671, Kingdom of Saudi Arabia; Consultant Anatomic Pathologist and Neuropathologist, Department of Pathology and Laboratory Medicine, King Faisal Specialist Hospital and Research Centre, Makkah Al Mukarramah Branch Road, Al Maather District, Riyadh 11211, Saudi Arabia

**Keywords:** H3K27M mutation, hemispheric glioma, diffuse midline glioma, adult brain tumor, high-grade glioma, neuro-oncology

## Abstract

The H3K27M genetic alteration is frequent in diffuse midline gliomas but rare in hemispheric diffuse gliomas. We report a rare case of a hemispheric H3K27M-mutant high-grade glioma in a 21-year-old female who presented with a seizure. Brain MRI revealed a 4.2 cm expansile left insular mass with cortical and subcortical involvement, consistent with a high-grade glial tumor. The patient underwent craniotomy and subtotal tumor resection. Microscopy demonstrated oligodendroglial-like morphology with microvascular proliferation and necrosis. Immunohistochemical analysis showed strong nuclear positivity for H3K27 M, IDH negativity, and loss of ATRX. Following progression on MRI in the left frontotemporal region, she underwent a redo craniotomy and debulking, which reproduced the previous histomorphology. The patient was subsequently referred for standard-of-care adjuvant chemoradiation, including external-beam radiation therapy with concurrent and adjuvant temozolomide, which represents the current recommended treatment approach for high-grade gliomas harboring H3K27M mutations. Additional therapeutic options, such as clinical trial enrollment, targeted agents, and immunotherapy, are increasingly considered for these aggressive tumors. This unusual hemispheric H3K27M-mutant tumor morphologically mimicked an oligodendroglioma. We recommend routine H3K27M testing in IDH-wildtype hemispheric gliomas with ATRX loss.

## Introduction

K27M mutations in H3F3A or HIST1H3 result in substitution of lysine with methionine at position 27 of histone H3, leading to loss of H3K27 trimethylation [1]. This alteration defines diffuse midline glioma (DMG), a WHO grade 4 entity that typically arises in midline structures and is associated with a poor prognosis, particularly in pediatric patients (median survival 9–15 months) [2].

Large-scale genomic studies have confirmed H3K27M mutations in both pediatric and adult high-grade gliomas and across diverse anatomical locations [3, 4]. Although classically described in children, adult patients may demonstrate more variable outcomes, suggesting that age, tumor location, and co-occurring molecular alterations influence prognosis [4, 5].

These findings underscore the importance of molecular profiling for accurate diagnosis and risk stratification, particularly in rare hemispheric presentations.

## Case report

A 21-year-old female with no known medical history presented with right arm numbness and a bloody taste in her mouth. This progressed to a right-sided headache, upward eye deviation, and right-sided clonic movements, followed by right-sided numbness and weakness. She was initially diagnosed with a generalized tonic–clonic (GTC) seizure and started on levetiracetam for seizure control, along with dexamethasone to reduce perilesional edema.

**Figure 1:**
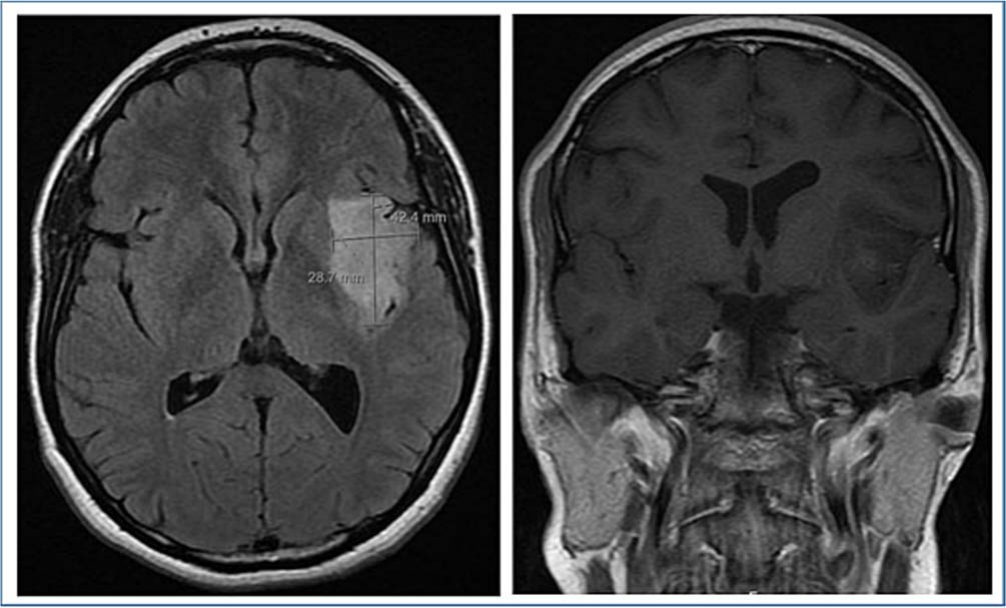
Preoperative brain MRI demonstrates a large, well-defined, expansile intra-axial mass lesion centered in the left insular region with cortical and subcortical involvement.

Magnetic resonance imaging (MRI) showed a 4.2 cm, well-defined, expansile intra-axial mass centered in the left insular region with cortical and subcortical involvement. The lesion demonstrated a central component of diffusion restriction and faint peripheral enhancement on post-contrast imaging (Fig. 1). The patient underwent a left frontotemporal craniotomy with subtotal (debulking) resection of the insular tumor. Postoperatively, she was doing well, with no headache, dizziness, nausea, vomiting, or signs of raised intracranial pressure.

**Figure 2:**
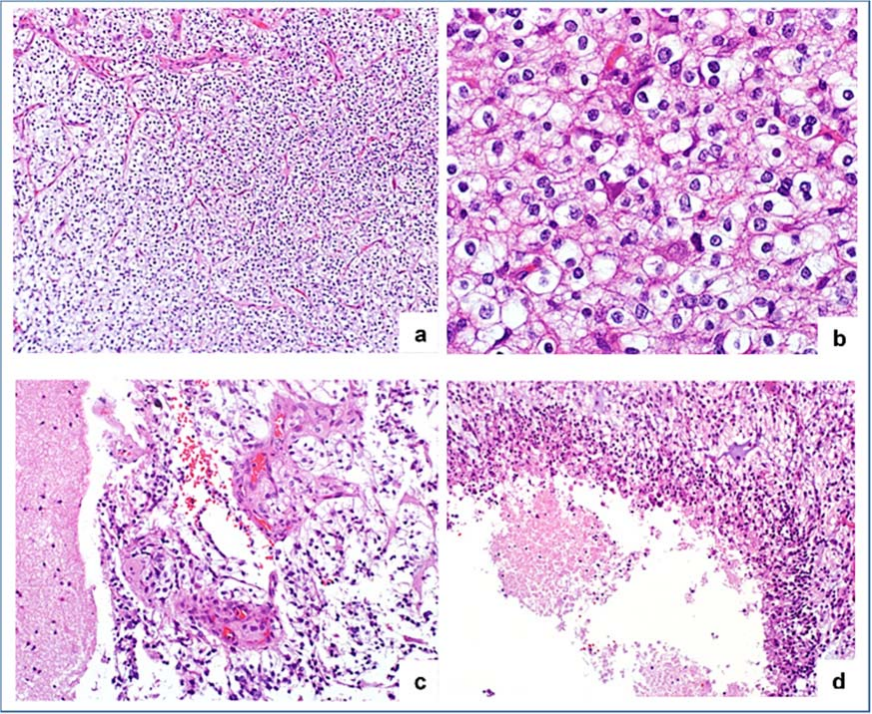
(a) and (b) the tumor morphology is predominantly oligodendroglial-like, with variable cellularity. (c) Area of microvascular proliferation. (d) Pseudo-palisading necrosis.

**Figure 3:**
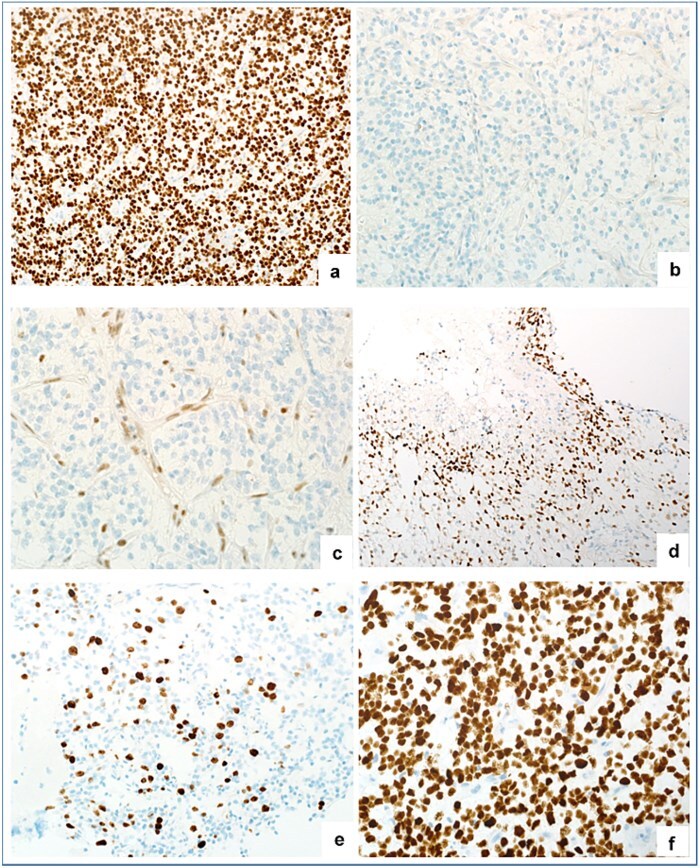
(a) Diffuse positivity of Olig2. (b) IDH-1 negative tumor cells. (c) Loss of ATRX staining. (d) p53 positivity. (e) Ki-67 proliferation index. (f) Diffuse nuclear positivity of H3K27M immunostain.

Histopathological evaluation revealed an infiltrative tumor with oligodendroglial-like morphology and variable cellularity predominantly from relatively low calcification (Ki-67 staining in this area was 5%) to high cellularity with focal microvascular proliferation and necrosis (Ki-67 staining in this area was 20%) (Fig. 2). Immunohistochemistry showed that the tumor cells were positive for GFAP, Olig2, and p53 expression, which varied from 5% to 15%. While negative for IDH R132H protein, CD34, BRAF, and ATRX showed complete loss of expression. To ensure completeness, H3K27M immunostaining was performed. The tumor cells showed strong, diffuse nuclear positivity (with appropriate negative nonneoplastic cells and a positive control performed simultaneously in a different case) (Fig. 3).

In this case, H3K27me3 immunostaining was unavailable. The differential diagnosis was an oligodendroglioma. It was excluded based on the immunohistochemical staining profile (loss of ATRX and positivity for p53), and the diffuse hemispheric H3G34 mutation was excluded based on the positivity of Olig-2 immunostaining. Oncomine NGS testing was positive for pathogenic mutations in H3-3A (p.(K27M), ATRX, and NF1, as well as amplification of CDK4, PIK3CA, and MDM2. The final pathological diagnosis was hemispheric high-grade diffuse glioma, H3K27M-mutant. Given the standard of care for H3K27-altered diffuse midline gliomas, which is focal radiotherapy (60 Gy in 30 fractions), the patient received postsurgical radiation. Concurrent and adjuvant temozolomide was administered, following the regimen commonly used in high-grade gliomas, to potentially improve tumor control by targeting rapidly dividing residual tumor cells and acting as a radiosensitizer during radiation. However, evidence for benefit in H3K27M-mutant tumors is limited. She completed 15 cycles of adjuvant temozolomide and continued to take antiseizure medications.

**Figure 4:**
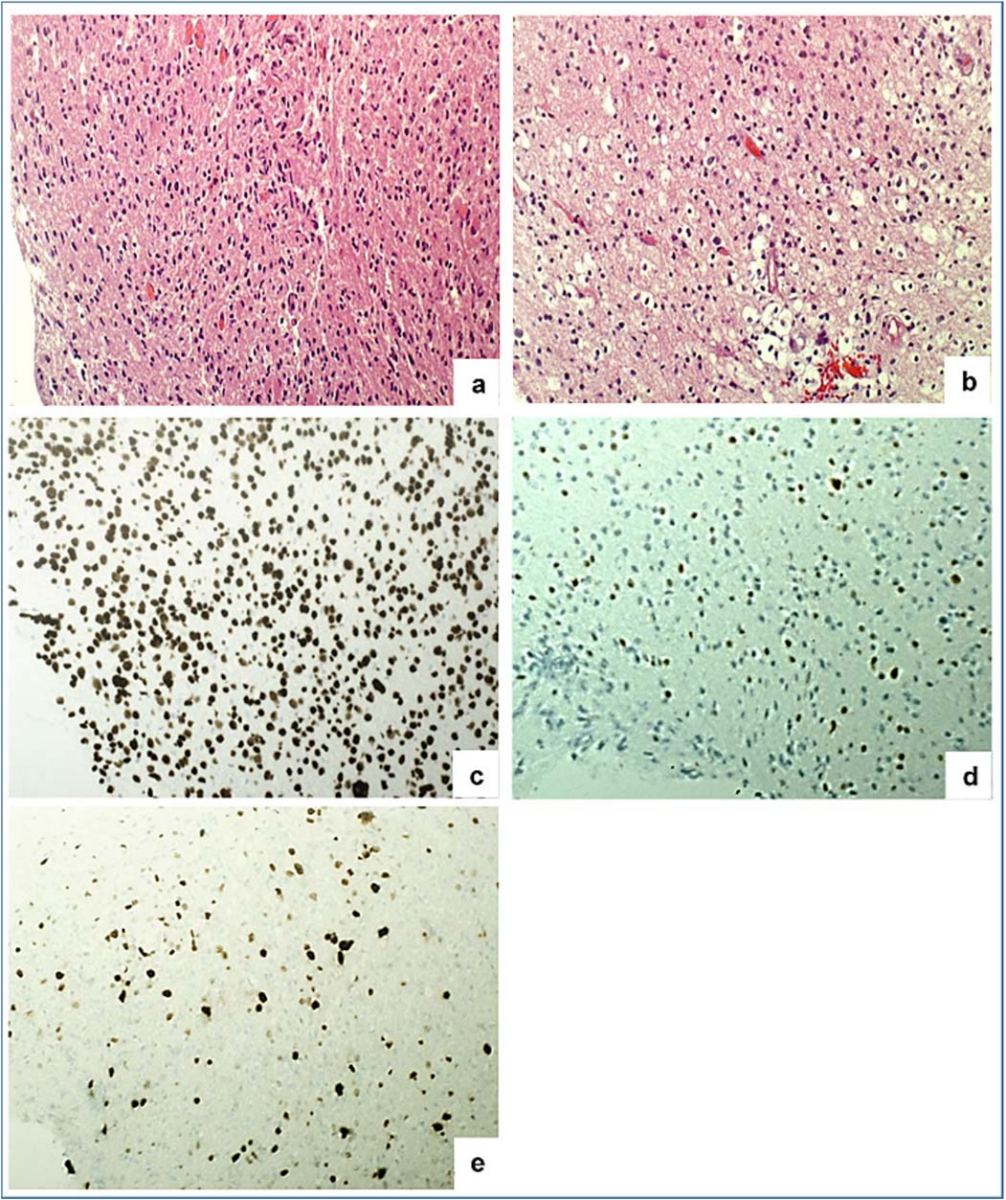
(a) Area of tumor with astrocytic morphology. (b) Area of tumor with oligodendroglial-like morphology. (c) Diffuse positivity of H3K27M. (d) Loss of ATRX staining in tumor cells. (e) Ki-67 proliferation index in > 20% of tumor cells.

Six months after the initial surgery, while still undergoing concurrent chemoradiotherapy and continuing antiseizure medications, she developed new episodes of electrical shock-like sensations on her left face, abnormal upper lip movements, and a bloody taste in her mouth. MRI demonstrated a progressive residual mass in the anterior pole of the left temporal lobe measuring 3.6 × 2.7 cm, with heterogeneous gadolinium enhancement, suggesting incomplete resolution of the primary tumor or early relapse rather than a true recurrence. She underwent a redo left temporal craniotomy with 5-aminovaleric acid-guided tumor debulking. Postoperative imaging following the second procedure was not available for review. Histologically, the tumor showed astrocytic and oligodendroglial-like morphologies (Fig. 4a and b). Immunohistochemistry repeatedly demonstrated intense, diffuse nuclear H3K27M staining, complete loss of ATRX, weak p53 staining, and a Ki-67 index of 20% (Fig. 4c–e). The findings were consistent with progressive residual diffuse hemispheric glioma, H3K27M-mutant rather than a true recurrence.

## Discussion

H3K27M alterations are characteristic of diffuse midline gliomas (DMGs) but are uncommon in hemispheric diffuse gliomas. While H3K27M-mutant DMGs are associated with poor survival, likely due in part to their location within critical midline structures where complete resection is rarely feasible [1], the prognostic significance of this mutation outside the midline remains unclear. Consequently, optimal management and risk stratification of nonmidline H3K27M-mutant gliomas have not been systematically defined.

To date, only nine cases of H3K27M-mutant hemispheric diffuse gliomas have been reported in the literature, including ours. Most occurred in adults (20–76 years), with a slight female predominance (5F:4M). Tumors were located in the frontal and temporal lobes (n = 3), insular cortex (n = 2), and corpus callosum (n = 4), with no evidence of midline involvement. Histologic grades ranged from 2 to 4 (grade 4: n = 5; grade 3: n = 3; grade 2: n = 1). All tumors were IDH-wildtype and confirmed H3K27M-mutant; ATRX loss was identified in three cases, including ours, while the remainder showed ATRX retention [4]. Recent cohort studies suggest that adults with H3K27M-mutant DMG have significantly better survival than pediatric patients (16 vs. 5 months; *P* = 0.0003) and may even outperform adults with H3-wildtype high-grade gliomas [5, 6]. These findings raise the possibility that the biological effect of the H3K27M mutation is context-dependent and may be modified by age, anatomical location, and co-occurring molecular alterations. A summary of previously reported adult hemispheric H3K27M-mutant diffuse gliomas is provided in Table 1.

**Table 1:** Summary of reported H3K27M-mutant hemispheric diffuse gliomas in adults.

Case	Age (yrs)	Sex	Tumor site	ATRX status	Outcome/follow-up	Reference
1	45	F	Frontal	Lost	12 mo	López et al. [1]
2	36	M	Temporal	Intact	14 mo	López et al. [1]
3	76	F	Corpus callosum	Lost	10 mo	López et al. [1]
4	20	M	Insular	Intact	18 mo	Chia et al. [4]
5	60	F	Corpus callosum	Intact	9 mo	Chia et al. [4]
6	50	F	Frontal	Lost	16 mo	Wang et al. [3]
7	28	M	Temporal	Intact	20 mo	Schulte et al. [5]
8	34	F	Insular	Intact	11 mo	Zheng et al. [6]
9	42	F	Temporal (anterior pole)	Lost	Alive at the latest follow-up	Present case

ATRX Status: Determined by immunohistochemistry or sequencing; ‘Lost’ indicates loss of ATRX expression. Outcome/Follow-up: Overall survival in months or patient status at last follow-up.

To contextualize hemispheric H3K27M-mutant tumors within the broader spectrum of H3K27M-driven gliomas, key distinctions between midline and hemispheric presentations are summarized in Table 2.

**Table 2:** Comparison of midline and hemispheric H3K27M-mutant diffuse gliomas.

Feature	Midline H3K27M-Mutant glioma (Diffuse Midline glioma, WHO Grade 4)	Hemispheric H3K27M-Mutant glioma
Typical age distribution	Predominantly pediatric; median survival ~ 9–15 months [2, 6]	More frequently reported in adults; survival variable and potentially longer than pediatric DMG [4–6]
Anatomical location	Brainstem (pons), thalamus, spinal cord; defined by midline involvement [1, 3]	Cerebral hemispheres (frontal, temporal, insular) and corpus callosum without classic midline origin [4]
WHO classification	Designated WHO grade 4 regardless of histologic features (WHO CNS 2021)	Not separately classified; grading based on histologic and molecular context
Surgical resection feasibility	Often limited due to eloquent midline structures and infiltrative growth [1]	Frequently feasible; subtotal or gross total resection may be possible
Molecular context	H3K27M is often the primary driver; ATRX loss variable	May co-occur with ATRX loss and other molecular alterations, suggesting context-dependent biology
Prognosis	Generally poor; aggressive clinical course [2, 6]	Prognostic significance is less clearly defined; it may be influenced by age, location, and co-alterations [5, 6]
Standard therapeutic approach	Fractionated radiotherapy is standard-of-care; limited systemic options	Radiotherapy remains standard-of-care; adjuvant chemotherapy is often considered in adults with resectable tumors

In our case, the tumor not only harbored an H3K27M mutation but also exhibited loss of ATRX expression, an uncommon combination in hemispheric tumors with oligodendroglioma-like morphology. The immunohistochemical profile and its relevance to the differential diagnosis are summarized in Table 3.

**Table 3:** Immunohistochemical markers and diagnostic implications.

Marker	Result in the present case	Diagnostic implication
IDH1 R132H	Negative	Excludes IDH-mutant astrocytoma and oligodendroglioma; supports IDH-wildtype high-grade glioma category
ATRX	Loss of nuclear expression	Strongly favors astrocytic lineage; argues against oligodendroglioma, which typically shows retained ATRX and 1p/19q co-deletion
p53	Focal nuclear positivity	Suggests TP53 pathway alteration; when combined with ATRX loss, supports astrocytic lineage over oligodendroglial tumors
Olig2	Diffuse nuclear positivity	Confirms glial origin; seen in both astrocytic and oligodendroglial tumors
H3K27M	Strong diffuse nuclear positivity	Defines H3K27M-mutant diffuse glioma subtype and indicates epigenetically driven tumor biology

This co-occurrence may have important biological and prognostic implications. ATRX is a chromatin-remodeling protein responsible for H3.3 incorporation at telomeric and heterochromatic regions. Its loss leads to widespread epigenetic dysregulation, disruption of chemical modifications that regulate gene expression without altering the DNA sequence, impaired DNA repair, and increased genomic instability [7, 8]. When combined with the H3K27M mutation, which inhibits the PRC2 complex, a histone-modifying complex responsible for H3K27 trimethylation and gene silencing, and globally reduces H3K27 trimethylation [2], the dual disruption may intensify epigenetic instability and drive a more aggressive, stem-like phenotype. ATRX loss also impairs chromatin remodeling and telomere maintenance, processes essential for genomic stability during cell division, contributing to replication stress and genomic instability [7, 8]. Together, these alterations promote tumor plasticity.

Based on emerging literature, ATRX loss may influence the biology of H3K27M-mutant hemispheric gliomas through three interconnected mechanisms:

1. Epigenetic dysregulation and dedifferentiation. ATRX loss disrupts chromatin remodeling and H3.3 deposition, while H3K27M inhibits PRC2, leading to reduced H3K27me3 and altered gene regulation [2, 8].
2. Genomic instability and treatment response. ATRX inactivation compromises DNA repair pathways, increasing replication stress and genomic instability [7].
3. Immune and microenvironmental modulation. ATRX deficiency affects heterochromatin regulation and the suppression of endogenous retroviral elements, potentially influencing immune-related gene expression [8].

Therapeutically, radiotherapy remains the standard of care for H3K27M-mutant gliomas. In adults with hemispheric tumors, temozolomide is often administered following resection. Investigational approaches include ONC201 [9] and immunotherapeutic strategies such as GD2-CAR T cells [10]. Circulating tumor DNA analysis may provide a non-invasive method for monitoring treatment response and disease progression [11].

Simplified Diagnostic Workflow for High-Grade Gliomas:

1. Clinical presentation and neuroimaging assessment (tumor location, enhancement pattern, mass effect).
2. Surgical resection or biopsy for histopathologic evaluation (cellularity, mitotic activity, necrosis, microvascular proliferation).
3. Immunohistochemical profiling (IDH1 R132H, ATRX, p53, Olig2, H3K27M).
4. Molecular confirmation by next-generation sequencing when indicated.

In IDH-wildtype hemispheric tumors, particularly those demonstrating ATRX loss, additional testing for H3K27M may prevent diagnostic misclassification.

Taken together, the co-occurrence of H3K27M mutation and ATRX loss in a hemispheric diffuse glioma may represent a biologically distinct subgroup with unique epigenetic and clinical behavior. This case underscores that the H3K27M mutation is not restricted to midline structures and may occur in IDH-wildtype hemispheric tumors with oligodendroglioma-like morphology. Comprehensive molecular testing, particularly in IDH-wildtype gliomas with ATRX loss, is therefore essential to prevent misclassification, refine prognostic stratification, and inform therapeutic decision-making. Larger molecularly characterized cohorts will be necessary to determine whether this dual-alteration phenotype confers distinct clinical outcomes compared with classic diffuse midline gliomas.
